# Editorial: Genetic Kidney Diseases of Childhood

**DOI:** 10.3389/fped.2018.00409

**Published:** 2018-12-19

**Authors:** Miriam Schmidts, Max C. Liebau

**Affiliations:** ^1^Genome Research Division, Human Genetics Department, Radboud University Medical Center Nijmegen and Radboud Institute for Molecular Life Sciences, Nijmegen, Netherlands; ^2^Center for Pediatrics and Adolescent Medicine, University Hospital Freiburg, Freiburg University Faculty of Medicine, Freiburg, Germany; ^3^Department of Pediatrics, University Hospital of Cologne, Cologne, Germany; ^4^Center for Molecular Medicine, University Hospital of Cologne, Cologne, Germany; ^5^Nephrology Research Laboratory, Department II of Internal Medicine, University Hospital of Cologne, Cologne, Germany

**Keywords:** ciliopathies, podocytes, nephrotic syndrome, cystic kidney disase, tubulopathies

Human kidneys are most spectacular organs. They have a busy life with filtering well over 100 liters of fluid every day and a few million liters over the course of a life. Not only is proper renal function essential for water and electrolyte balance and excretion of toxic substances in mammals, but our kidneys have amazing additional roles regarding blood pressure regulation and stimulation of red blood cell renewal. Those diverse functions are achieved by an array of different renal cell types, forming a complex tissue architecture. The “building plan” ensuring formation and maintenance of these amazing organs lies within our genes and tiny changes within this renal map will have devastating consequences within this fine-tuned building of blood vessels, glomeruli, and tubules. It therefore comes to no surprise that in children and adolescents, genetic defects are the most common cause for end stage renal disease.

Over the last years there has been outstanding progress in the knowledge about genetic kidney diseases, including the identification of multiple disease-associated genes and insights into the cellular pathophysiology. These developments have profoundly changed our understanding of genetic kidney diseases and our therapeutic approaches to (pediatric) patients suffering from these disorders. In this research topic on Genetic Kidney Diseases of Childhood published in *Frontiers in Pediatrics* we aim to give the reader interested in pediatric nephrology a broad overview over a variety of genetic kidney diseases and recent developments in clinical fields, from a research point of view as well as from a patient's perspective.

Amongst the most common heriditary renal disorders with childhood onset are glomerular diseases. The discovery of genetic origins of steroid resistant nephrotic syndrome has provided great diagnostic progress and dramatically influenced the therapeutic pathway for affected families, protecting affected children from unnecessary and unsuccessful immunosuppressive treatments (Kemper and Lemke). Further, genetic research in combination with cell biology and biochemistry approaches has revealed multiple novel components of the glomerular filtration barrier, greatly improving our biological understanding in general (Hagmann and Brinkkoetter). Novel experimental approaches including work on model organisms like *Drosophila melanogaster* or *Danio rerio* as described in two manuscripts of the research topic have further contributed to unravel the molecular mechanisms resulting in the clinical presentation of nephrotic syndrome and the histology of e.g., focal and segmental glomerulosclerosis (Helmstädter et al.; Gehrig et al.).

The observational Podonet-approach follows a large international cohort of patients and describes genotype-phenotype correlations (Trautmann et al.). This study has interestingly revealed that changes in collagen genes may underly a substantial number of patients with focal and segmental glomerulosclerosis as also described by an independent group (Braunisch et al.). While podocyte-focussed research has received a lot of attention during the last years, genetic defects of the glomerular basement membrane thus represent a substantial cause for glomerular disease (Chew and Lennon).

Another rapidly-evolving field in genetic kidney diseases deals with cystic kidney diseases. This group of disorders is important for both children and adults and includes autosomal dominant and autosomal recessive polycystic kidney disease, nephronophthisis, and various rare syndromes presenting with cystic kidneys including e.g., Bardet-Biedl syndrome (BBS), Meckel Gruber syndrome (MKS), or von Hippel Lindau syndrome (VHL).

The management of pediatric patients with cystic kidney diseases remains a clinical challenge as no causative treatment options are available to date. The research topic at hand discusses some important aspects and presents state-of-the-art knowledge, including current and potential future ways of managing BBS (Hartill et al.), MKS (Forsythe et al.), or VHL (Kim and Zschiedrich), approaches to diagnosis and treatment of cystic kidney diseases in adults (Müller and Benzing), and current opinions on the use of gastrostomy tube insertion in children with ARPKD (Burgmaier et al.). A topic of ongoing debate is the question whether children of patients suffering from ADPKD should undergo early diagnosis or not. These children are at a 50% risk of having inherited the genetic variants responsible for ADPKD, a disorder that typically develops over decades. It has not yet been fully established whether children benefit from early diagnosis or whether the e.g., psychosocial burden of an early diagnosis outweighs benefits. Two manuscripts in the research topic deal with different aspects of ADPKD in children (De Rechter et al.; Harris). The genetic and cellular changes underlying ADPKD are summarized in an additional manuscript (Cordido et al.).

Over the last years a lot of attention has been paid to a specific cellular organelle, whose dysfunction has been linked to the development of cystic kidney disease. This organelle is the primary cilium, an antennae-like structure on the cellular surface that seems to be involved in the sensing of the extracellular environment. As cilia can be found on multiple cell types, it is plausible that cystic kidney diseases frequently present with extrarenal manifestations as in nephronophthisis and nephronophthisis-associated diseases. A manuscript of this research topic summarizes the molecular mechanisms and the genetic basis resulting in Nephronophthisis (Srivastava et al.). Two additional manuscripts describe current collaborative research consortia that aim to link the findings of genetics and cellular biology with a deep clinical phenotyping and biobanking in order to set the basis for evidence-based clinical recommendations and future translational research approaches for cystic kidney diseases (König et al.; Renkema et al.).

Likewise, an array of defective genes has been identified to date to cause renal tubular dysfunction, leading to diverse phenotypes in human such as Bartter syndrome (Yang et al.) Cystinosis (Bäumner and Weber), magnesium transport defects (Giménez-Mascarell et al.) or inherited disorders with kidney stone formation (Halbritter et al.). Genetic diagnosis has not only provided opportunities of highly specialized clinical care and genetic counseling of at-risk and carrier individuals but also offers for the first time causative treatment options such as for cystinosis where cysteamine therapy has now been implemented.

Inherited renal diseases with childhood onset can be manifestations of syndromal disease patterns with multiple organ systems involved such as in case of ciliopathies like BBS or MKS. Other examples include rare conditions affecting the skin and the kidneys (Reimer et al.), resulting from basal membrane defects or defects involving the heart and the kidneys (Gabriel et al.) The latter often but not always result from cilia dysfunction, however the precise underlying molecular mechanisms, e.g., cell signaling pathways defective, have not been understood. Last but not least, patients with congenital anomalies of the kidney and urinary tract (CAKUT) can present with complex syndromal appearances as described for individuals harboring PBX1 mutations (Riedhammer et al.) and kidney function seems to be regulated by programming events very early in life that likely result in long-term modulation of gene function in the kidney (Nüsken et al.).

This research topic provides a concise overview about current state-of-knowledge and outlook on future developments with respects to the diverse landscape of inherited childhood onset renal diseases.

## Author Contributions

All authors listed have made a substantial, direct and intellectual contribution to the work, and approved it for publication.

### Conflict of Interest Statement

ML has received honoraria for scientific lectures from Pfizer. Representing the University Hospital of Cologne, ML has been counseling Otsuka in an advisory board. The remaining author declares that the research was conducted in the absence of any commercial or financial relationships that could be construed as a potential conflict of interest.

